# Rational design of modular circuits for gene transcription: A test of the bottom-up approach

**DOI:** 10.1186/1754-1611-4-14

**Published:** 2010-11-11

**Authors:** Francesca Ceroni, Simone Furini, Emanuele Giordano, Silvio Cavalcanti

**Affiliations:** 1 Laboratory of Cellular and Molecular Engineering, University of Bologna, I-47521 Cesena, Italy; 2 Department of Electronics, Computer Science and Systems, University of Bologna, I-47521 Cesena, Italy; 3 Department of Medical Surgery and Bioengineering, University of Siena, I-53100 Siena, Italy; 4 Department of Biochemistry "G. Moruzzi", University of Bologna, I-40126 Bologna, Italy

## Abstract

**Background:**

Most of synthetic circuits developed so far have been designed by an ad hoc approach, using a small number of components (i.e. LacI, TetR) and a trial and error strategy. We are at the point where an increasing number of modular, inter-changeable and well-characterized components is needed to expand the construction of synthetic devices and to allow a rational approach to the design.

**Results:**

We used interchangeable modular biological parts to create a set of novel synthetic devices for controlling gene transcription, and we developed a mathematical model of the modular circuits. Model parameters were identified by experimental measurements from a subset of modular combinations. The model revealed an unexpected feature of the lactose repressor system, i.e. a residual binding affinity for the operator site by induced lactose repressor molecules. Once this residual affinity was taken into account, the model properly reproduced the experimental data from the training set. The parameters identified in the training set allowed the prediction of the behavior of networks not included in the identification procedure.

**Conclusions:**

This study provides new quantitative evidences that the use of independent and well-characterized biological parts and mathematical modeling, what is called a bottom-up approach to the construction of gene networks, can allow the design of new and different devices re-using the same modular parts.

## Background

Synthetic biology has evolved to the point where the design of gene circuits with complex functionalities has become a real option [1]. Inside living cells, complex behaviors arise from molecular interplays in complicated regulatory networks. In the first instance, the ability to isolate single elements from these regulatory networks - and to use them as independent modules - makes synthetic biology possible [2]. Synthetic gene circuits are thus created by assembling elementary modules together. The increasing complexity of these synthetic gene networks asks for a rational approach to design gene circuits [3]. A possible strategy to tackle this complexity is the *bottom-up *approach [4-7]. In bottom-up design, the behavior of a complicated system is predicted from the characteristics of its elementary parts. Such a prediction requires well-characterized mathematical models of these elementary parts, and of how they behave when assembled together. In the present study, we tested if it is possible to predict by mathematical modeling the behavior of a modular gene circuit, using as inputs the properties of its elementary parts.

In the last decade, many elementary devices have been implemented both in prokaryotic and in eukaryotic cells [8-11], including logical gates [12], toggle switches [13], oscillators [14,15], band pass filters [14] and counters [16]. However, most of the circuits developed so far have been designed by an *ad hoc *approach, using specific gene components and a trial and error strategy. In order to make the design of synthetic systems easier, great efforts are today directed to extend to synthetic biology the engineering concepts of standardization, modularity, and abstraction [17,18]. In this context, the Registry of Standard Biological Parts http://www.partsregistry.org, maintained by the Massachusetts Institute of Technology (MIT), distributes thousands of standard parts, named BioBricks. The BioBricks are only standardized in terms of how these individual parts can be physically assembled into more complicated systems, whilst most of them remain not tested in terms of their functionality and biological behavior [19].

The option to use modular parts in large-scale networks will be highly facilitated by a detailed characterization of their functional properties shared by the synthetic biology community. This implies the standardization of tools, techniques, and measurement units used by different laboratories [19-21], and the definition of mathematical models for the single parts. In this direction, a repository of modular modeling components has recently been published to facilitate the mathematical modeling of biological parts, and to help the design process in synthetic biology [22]. Once mathematical models of the elementary components are available, the following step is to use them to predict the behavior of larger-scale networks. Only if the behavior of gene circuits can be predicted from the properties of their elementary parts, a bottom-up design of synthetic devices is feasible [23]. While this remains a central problem of synthetic biology, few quantitative tests of how modular systems behave have been published. One example is the work by Ellis et al [24] where a library of regulated promoters is characterized and used to design more complicated networks with predictable outcomes. This quantitative study supports the case of a bottom-up approach to gene circuit design.

**Figure 1:**
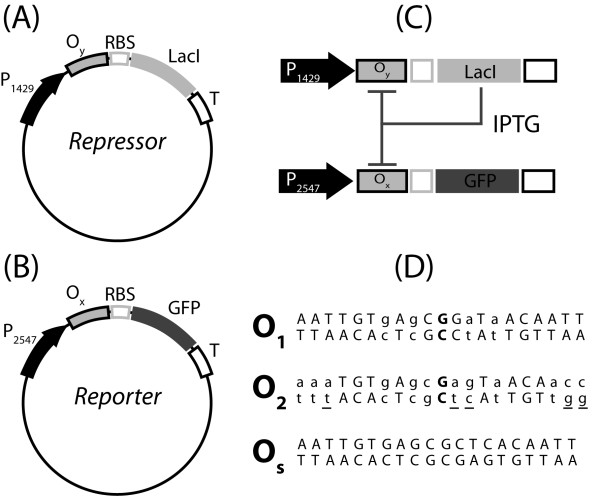
**Gene circuits**. *Repressor *(A) and *Reporter *(B) plasmids. Boxes highlight the parts that could be used as independent elements to tune the circuit outcome in a modular framework. As a proof of principle of the modular architecture, we created nine different gene circuits, combining three different operator sequences, O_1_, O_2 _and O_s_, in two plasmids. (C) Gene circuit in cells transformed with both the plasmids. (D) Sequences of the three operator sites. Bold characters are used for the central G-C base pair in the naturally occurring operator sites O_1 _and O_2_. The left and right half operator sites in O_1 _and O_2 _are pseudo-symmetric, lower-case characters highlight the deviations from perfect symmetry. The base pairs that are different between the operators O_1 _and O_2 _are underlined in the O_2 _sequence. The artificial operator site O_s _was defined removing the central base pair G-C and creating a perfectly symmetric sequence based on the left half of the operator O_1_. The affinity to lactose repressor of the three operator sites O_1_, O_2 _and O_s _spans a range of over two orders of magnitude [27,28], which allows the analysis of the system behavior under wide changes of the parameters values.

In this study we used, as a prototypal model, a synthetic device based on the lactose repressor system. This synthetic gene network includes: (i) a negative feedback circuit for the synthesis of lactose repressor (LacI) molecules; (ii) and a reporter circuit, for the synthesis of green fluorescent protein (GFP) controlled by LacI (Figure 1). Transcriptional control is realized by regulated promoters, assembled from a constitutive promoter and an operator site docking for LacI. The prokaryotic promoter can be divided into three sub-regions: (i) distal (upstream of the -35 sequence), (ii) core (between -35 and -10) and (iii) proximal (downstream of the -10) [25]. LacI can regulate gene transcription by binding to operator sites placed in any of these regions [26]. However, we decided to insert our operator sites only into the proximal promoter region, avoiding modifications of the integrity of the core region. This choice preserves the basal transcriptional level of the promoter, and most importantly its modularity. The operator sites in the synthetic devices were used as modular elements. Nine different devices were assembled using three operator sites with different binding affinities for LacI, alternatively inserted into two plasmid vectors. Three of these synthetic circuits were used to define the mathematical model, and to identify its parameters. The mathematical model was then used to predict the behavior of the remaining untested circuits, providing a quantitative test of modular design in synthetic biology.

## Results

### Definition of the gene-circuits

*E. coli *DH5α cells were transformed with two different plasmids, here named *Repressor *and *Reporter *(Figure 1). The gene circuit encoded in the *Repressor *plasmid has a negative feedback structure as an operator site for LacI is inserted downstream of a promoter sequence controlling the expression of the lactose repressor gene itself (Figure 1A). The concentration of LacI in the cytoplasm also controls the transcription of the GFP gene on the *Reporter *gene circuit (Figure 1B). The synthesis of GFP in a cell transformed with both *Repressor *and *Reporter *plasmids can be tuned by changing the concentration of the gratuitous inducer IPTG (Figure 1C). We created nine different gene circuits, using all the possible combinations of three operator sites (O_1_, O_2 _and O_s_, see the sequences in Figure 1D) in the *Repressor *and *Reporter *plasmids. The affinity to lactose repressor of the operator sites O_1_, O_2 _and O_s _spans a range of over two orders of magnitude, which allows the creation of systems with widely different characteristics [27,28]. The symbol O_x_O_y _is used to identify the gene circuit with the operator sequence O_x _on the *Reporter *plasmid, and the operator sequence O_y _on the *Repressor *plasmid. If cells are transformed with the *Reporter *plasmid alone, the symbols O_1_, O_2_, or O_s _are used, according to which operator site is present.

### Test of the modular architecture

**Figure 2:**
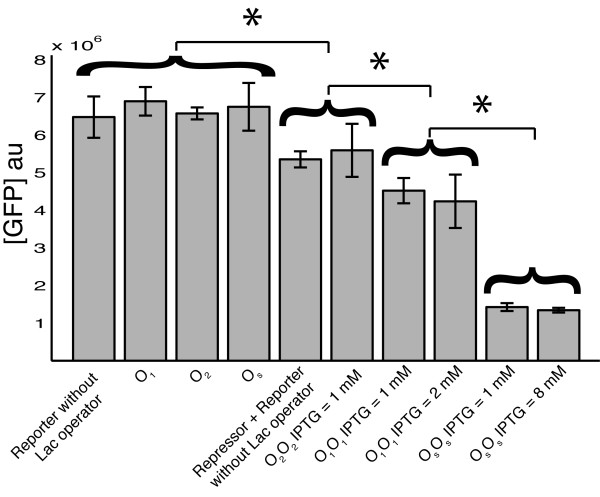
**Fluorescence levels**. The normalized fluorescence, equation 2, in arbitrary units, is shown for cells transformed with: *Reporter *plasmid without the operator sequence (n = 6); *Reporter *plasmids with the O_1 _(n = 5), O_2 _(n = 8), and O_s _(n = 10) operator sequence; *Repressor *plasmids with any of the three operator sequences and *Reporter *plasmid lacking the lactose operator sequence (n = 10); O_2_O_2 _induced with 1 mM IPTG (n = 13); O_1_O_1 _induced with 1 mM (n = 9) and 2 mM (n = 5) IPTG; O_s_O_s _induced with 1 mM (n = 4) and 8 mM (n = 3) IPTG. In absence of *Repressor *plasmids, the normalized fluorescence is the same if the *Reporter *plasmid does not include the operator sequence, or if it includes any of the three operator sequences tested. Co-transformation with a *Repressor *plasmid and a *Reporter *plasmid without the operator site causes a decrease in the maximum fluorescence, even if no LacI activity is exerted. The same fluorescence was observed in the O_2_O_2 _gene circuit at saturating concentrations of IPTG. On the other hand, the maximum fluorescence in the O_1_O_1 _and O_s_O_s _gene circuits is statistically different from the maximum value.

Cells transformed only with *Reporter *plasmids showed equilibrium values of the normalized fluorescence equal to (see the Methods section for the definition of normalized fluorescence, [*GFP*]*au*) 6.89 × 10^6^, 6.55 × 10^6 ^and 6.75 × 10^6 ^arbitrary units (au) for plasmids with the O_1_, O_2 _and O_s _operator sequence respectively (Figure 2). In absence of *Repressor *plasmids, no molecules of LacI are synthesized, thus no LacI-dependent inhibition is exerted on GFP transcription. In accordance, cells transformed with *Reporter *plasmids with different operator sequences yielded analogous fluorescence levels. This result supports the case of a modular architecture of the gene circuit, where promoter and operator sequences can be used as independent modular parts. In agreement, cells transformed only with a *Reporter *plasmid lacking the operator sequence showed equilibrium fluorescence of 6.47 × 10^6 ^au (Figure 2). This value is statistically equivalent to those measured in cells transformed with a *Reporter *plasmid containing one of the three operator sequences. This suggests that the presence of the operator sequence alone does not affect transcription rates. Thus, a unique value of the GFP transcription rate, $\alpha_{G}^{M}$, was used in the mathematical model, independently of the specific operator sequence present in the gene circuit (see the Methods section for the definition of the mathematical model, and Table 1 for the list of the parameters).

**Table 1:** Variables and parameters of the mathematical model.

	Description		Value	Units
*I*	IPTG concentration			mM

*G*	Concentration of GFP protein			molecules per cell

*L*^*F*^	Free LacI molecules, i.e. not bound to operator sites or IPTG molecule			molecules per cell

*L*^*I*^	LacI molecules bound to IPTG			molecules per cell

*M*_*G*_	mRNA molecules of GFP			molecules per cell

*M*_*L*_	mRNA molecules of LacI			molecules per cell

$D_{G/L}^{F}$	Free *Repressor/Reporter *plasmids			plasmids per cell

$D_{G/L}^{L}$	*Repressor/Reporter *plasmids bound to LacI molecules			plasmids per cell

$D_{G/L}^{I}$	*Repressor/Reporter *plasmids bound to induced LacI molecules			plasmids per cell

$D_{G}^{0}$	Number of *Reporter *plasmids per cell		80	plasmids per cell

$D_{L}^{0}$	Number of *Repressor *plasmids per cell		$D_{G}^{0}/3.75^{§}=21.33$	plasmids per cell

*λ*_*G/L*_	Protein degradation rate		0.0214^§^	min^-1^

$\lambda_{G/L}^{M}$	mRNA degradation rate		0.271[39]	min^-1^

*α*_*G*_	GFP rate of synthesis		540*λ*_*G *_= 11.54[40]	min^-1^

*Α*_*L*_	LacI rate of synthesis		*α*_*G*_/4 = 2.88	min^-1^

$\alpha_{G}^{M}$	GFP transcription rate		0.56^†^	min^-1^

$\alpha_{L}^{M}$	LacI transcription rate		$\alpha_{G}^{M}/1.23^{§}=0.45$	min^-1^

$K_{x}^{L}$	Equilibrium binding constant of the complex LacI-O_x_	$K_{1}^{L}$	0.13[41]	molecules per cell
		
		$K_{2}^{L}$	1.63[41]	molecules per cell
		
		$K_{s}^{L}$	0.0394^†^	molecules per cell

$K_{x}^{I}$	Equilibrium binding constant for the binding of induced LacI molecule to the operator sequence O_x_	$K_{1}^{I}$	25336^†^	molecules per cell
		
		$K_{2}^{I}$	∞	molecules per cell
		
		$K_{s}^{I}$	313^†^	molecules per cell

*K*^*LI*^	Equilibrium binding constant for the binding IPTG-LacI		0.2890^†^	mM

*n*	Cooperativity of the binding LacI-IPTG		1.8688^†^	

*τ*^*LI*^	Time constant of LacI binding to the operator sequences		0.02[43]	min

*τ*^*DI*^	Time constant of induced-LacI binding to the operator sequences		*	min

*τ*^*DL*^	Time constant of the binding LacI-IPTG		*	min

* Only the steady-state behaviors of the gene circuits are analyzed, thus arbitrary values can be used for these time constants. ^†^Values defined through the fitting procedure. ^§^Values obtained by experimental measurements. References are included for the values retrieved from the literature.

Cells transformed with the *Reporter *circuit alone reached the maximum fluorescence level in our experimental conditions. In an ideal modular system, this maximum fluorescence is conserved in cells transformed with both *Reporter *and *Repressor *plasmids, if the *Reporter *plasmids lack the lactose operator sequence. To test this issue, we co-transformed cells with a *Repressor *plasmid, containing any of the three different operator sites, and with a *Reporter *plasmid lacking the operator site. Cells co-transformed with these two plasmids (*Repressor *plasmid and *Reporter *plasmid lacking the operator site) yielded a fluorescence value of 5.44 × 10^6 ^au, significantly lower than the maximum fluorescence observed in cells transformed with either of the *Reporter *plasmids alone (Figure 2). The average fluorescence in cells co-transformed with the *Repressor *plasmid and the *Reporter *plasmid without the operator site, was used to define the maximum fluorescence level of the gene-circuit (*GFP*_max_). The relative fluorescence, *GFP*_%_, in the gene circuits O_x_O_y_, was defined as:

(1) $$GFP_{\%}=\frac{[GFP]au}{GFP_{\max}}$$

Only in the O_2_O_2 _gene circuit a relative fluorescence close to 100% was obtained upon IPTG induction. Indeed, the fluorescence observed in the O_2_O_2 _gene circuit in presence of 1 mM IPTG was not significantly different from the maximum fluorescence produced by the gene circuit with the *Repressor *plasmid and the *Reporter *plasmid without the operator site. On the other hand, fluorescence values induced by 1 mM IPTG in the O_1_O_1 _and O_s_O_s _gene circuits were statistically different from *GFP*_max_. Increasing the IPTG concentration up to 2 mM in O_2_O_2_, and up to 8 mM in O_s_O_s_, did not change the fluorescence value, suggesting that the removal of LacI-mediated inhibition of transcription is already saturated at 1 mM IPTG in both the gene circuits (Figure 2).

**Figure 3:**
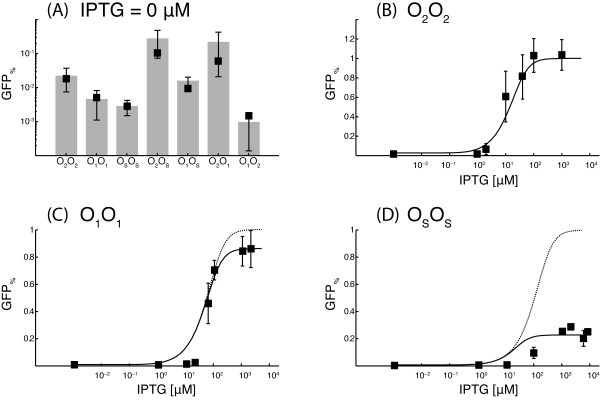
**Percentage fluorescence in absence of IPTG (A), and dose-response curves for the gene-circuits O**_**2**_**O**_**2 **_**(B), O**_**1**_**O**_**1 **_**(C), and O**_**s**_**O**_**s **_**(D)**. In panel (A) grey bars with standard deviations are used for the experimental data while black squares are used for the simulated responses. In panels (C) and (D), a continuous line is used for the simulated dose-response curves in presence of a residual affinity for the operator sites by induced LacI molecules, while a dotted line is used for the simulated dose-response curves when this residual affinity is turned off. The y-axis is in logarithmic scale in panel (A). The x-axis is in logarithmic scale in panels (B) to (D). All the measurements were repeated at least five times.

In absence of IPTG, the gene-circuits O_2_O_2_, O_1_O_1 _and O_s_O_s _showed relative inductions of 2.22 × 10^-2^, 4.62 × 10^-3 ^and 2.85 × 10^-3 ^respectively (Figure 3A). The relative inductions of the three gene circuits with the same operator sequence on both plasmids were used to identify the model parameters. A single parameter, $\alpha_{G}^{M}$, was used in the fitting procedure of the gene circuits O_2_O_2 _and O_1_O_1_. The excellent agreement with the experimental data suggests that the mathematical model correctly reproduce the behavior of the gene networks in absence of IPTG (Figure 3A). A second parameter, $K_{s}^{L}$, was used to fit the data of the O_s_O_s _gene circuit, thus, it is not surprising that the mathematical model well reproduces this data.

To test the validity of the modular approach, selected combinations of *Reporter *and *Repressor *plasmids with different operator sites were tested, namely O_2_O_s_, O_1_O_s_, O_2_O_1_, and O_1_O_2_. The remaining combinations were discarded because the operator site in the *Reporter *plasmid was too strong compared to the operator site in the *Repressor *plasmid. The mathematical model predicts well the relative inductions of these four gene circuits in absence of IPTG (Figure 3A), with no further adjustment of the model parameters. The predictions of the model are closer than one standard deviation from the experimental data for all the gene circuits.

The dose-response curves of the gene circuits O_2_O_2_, O_1_O_1 _and O_s_O_s _were fitted using five parameters. Two parameters, *K*^*LI *^and *n*, describe the binding of IPTG to LacI and are the same for the three gene circuits. The other parameters, $K_{2}^{I}$, $K_{1}^{I}$ and $K_{s}^{I}$ describe the binding of induced LacI molecules to the respective operator sites. Since the gene circuit O_2_O_2 _reaches 100% induction at saturating concentration of IPTG, the parameter $K_{2}^{I}$ can be considered equal to infinite for any practical reason. On the other hand, the maximum fluorescence in the gene circuit O_1_O_1 _is statistically different from *GFP*_max_. In absence of LacI molecules, gene circuits with the O_1 _or O_2 _operator sequence on the *Reporter *plasmids showed the same fluorescence. Thus, the diminished maximum fluorescence of the O_1_O_1 _gene circuit can only result from a LacI-dependent impairment of transcription, persisting at saturating concentration of IPTG. A marginal affinity of the induced LacI, *L*^*I*^, to the operator sequence can explain the experimental data of the O_1_O_1 _gene circuit. Figure 3C shows the simulated dose-response curve if induced LacI molecules have (continuous line), or have not (dotted line) a residual affinity for the operator sites. Introducing the marginal affinity $K_{1}^{I}$ decreases the distance from the experimental data from 0.33 to 0.25. The effect of this marginal affinity is much more evident in the O_s_O_s _gene circuit, where only ~25% of GFP synthesis was achieved at saturating concentrations of IPTG (Figure 3D). According to the estimated parameters, the operator sites O_1 _and O_s _bind the free LacI molecules respectively ~2 × 10^5 ^and ~1 × 10^4 ^times stronger than the induced LacI molecules.

**Figure 4:**
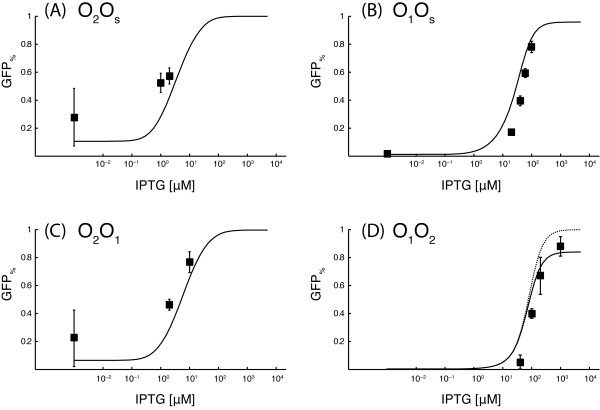
**Dose-response curves for gene circuits with different operator sequences on the *Reporter *and the *Repressor *plasmids**. A continuous line is used for the simulated dose-response curves in presence of a residual affinity for the operator sites by induced LacI molecules, while a dotted line is used for the simulated dose-response curves when this residual affinity is turned off. All the measurements were repeated at least five times.

The predicted dose-response curves for the O_2_O_s_, O_1_O_s_, O_2_O_1 _and O_1_O_2 _gene circuits are in reasonable agreement with the experimental data (Figure 4). It is important to remark that the experimental data for these four circuits were not used in the fitting procedure, and thus the simulated curves in Figure 4 have to be intended as purely predictive.

## Discussion

The design of new synthetic gene devices will be highly facilitated by a modular structure of gene circuits and a bottom-up strategy. While the synthetic biology community is rapidly adopting a modular architecture, quantitative studies of how multi-part systems behave are still missing. Here, we analyzed gene transcription in a modular gene circuit, and tested if the properties of the complete device can be predicted from the properties of its elementary parts. The major findings are that: (i) each modular part may behave in a different way if it is either isolated or included in a more complex gene circuit; and (ii) mathematical modeling can, however, predict the outcome of the device, if the elementary parts are characterized in the proper experimental conditions. These experimental conditions must be as close as possible to the gene circuit where the elementary parts should be used, in order to compensate for the non-ideal modularity of the system.

In an ideal modular system, the insertion of a new component should not affect the properties of the other parts, as each of them should work independently. In the real world, this is never the case. The properties of the different parts in a biological system are obviously affected by other parts. This poses major problems for the design of synthetic gene circuits with a bottom-up approach. Even the simplified system analyzed in this study is not strictly modular. In fact, cells transformed with both the *Repressor *and the *Reporter *plasmids have lower fluorescence than those transformed with the *Reporter *plasmid alone, even though the LacI-dependent repression of gene transcription is completely suppressed by removing the operator sequence from the promoter. The decreased fluorescence in cells transformed with both the plasmids is likely due to the extra burden that the cells have to sustain in presence of a second plasmid and antibiotic [29,30]. Since the same synthetic device can have different outcomes depending on the gene transcription rates, the deviations from a perfect modular behavior have to be carefully taken into account in the design of new gene circuits. Thus, if we want to use a bottom-up approach we need to characterize the elementary parts in a gene circuit reproducing to the closest extent the final synthetic device.

Here we characterized the elementary parts measuring fluorescence in cells transformed with *Reporter *and *Repressor *plasmids having the same operator sequence and then we used the properties of these systems to predict the behavior of gene circuits with different operator sequences in the two plasmids. The analysis of the response of the circuits with the same operator in the *Reporter *and in the *Repressor *revealed an unexpected feature about the functionality of LacI. Experimental data are well reproduced by the mathematical model only if we hypothesize a residual affinity for the operator sites by the induced LacI molecules. This possibility has already been proposed [31,32], but to the best of our knowledge never measured experimentally. A direct measure of this residual affinity is hampered by its low value compared to the affinity of the operator for the free LacI molecules (4 and 5 orders of magnitude higher respectively for the O_s _and O_1 _operator sequences). Our data provide a first experimental measurement of these residual affinities in *E. coli*, which can be useful in mathematical modeling of the lactose repressor system.

An alternative explanation for the decrease of the maximum fluorescence in the O_1_O_1 _and O_s_O_s _gene circuits is the saturation of the intracellular IPTG concentration at a value around 1 mM. However, no data to support this hypothesis are present in literature for such a value of IPTG concentration. Indeed, IPTG can move through cell membranes both by a diffusive and by an active transport [33], the latter being more efficient for the inward motion. Under these conditions, the intracellular concentration of IPTG should be equal, if not higher, than the extracellular concentration.

The blockade of gene transcription by induced LacI molecules was essential to reproduce the non-complete induction of the O_1_O_1 _and O_s_O_s _circuits. When this term is included in the mathematical model, the theoretical dose-response curves of the three circuits with *Reporter *and *Repressor *plasmids having the same operator sequence are in agreement with the experimental data. Small deviations are observed for the O_s_O_s _circuit. Here, the computed IPTG concentration giving half the maximal fluorescence is lower than the experimental value. This discrepancy may result from a poor determination of the O_s_O_s _fluorescence in absence of IPTG. Due to the high affinity of LacI to the O_s _operator site, the fluorescence in absence of IPTG is close to cellular auto-fluorescence. Thus, its experimental value can be measured only with low accuracy, which is reflected in a poor determination of the parameter $K_{s}^{L}$ of the mathematical model. Lower values of $K_{s}^{L}$ give a better fitting of the raising phase of the dose-response curve of the O_s_O_s _circuit. However, finding the best fitting of the experimental data is out of the scope of this study. More detailed models of the lactose repressor system have been proposed [34,35] and these models may likely provide a better agreement to the experimental data. The choice of an extremely simple model was inspired both by practical and theoretical considerations. First, more detailed models have a higher number of parameters. If these parameters have to be assigned in a non-arbitrary way, more experimental measurements are needed. An excessive burden in the characterization of the elementary parts will make a bottom-up approach to gene circuit design not efficient. Second, mathematical models with many parameters can suffer from the over-fitting problem, i.e. the experimental data used in the fitting procedure are well reproduced because the model describes even the noise in the experimental data. If over-fitting occurs, the parameters of the model cannot be used to analyze slightly different systems. Since this is exactly what we wanted to do here, we preferred to use a simple mathematical model with fewer parameters.

In this context, the agreement between the theoretical and the experimental data of the O_1_O_s_, O_2_O_s_, O_2_O_1 _and O_1_O_2 _gene circuits is remarkable. The agreement is extremely good in absence of IPTG, while small deviations are observed in the dose-response curves. Even if the adopted circuit is simple, it allows the study of an important problem in synthetic biology, i.e. the regulation of gene transcription. In fact, quantitative control of gene expression has always been a central problem for the design of gene networks. While designing the logical scheme of a network is relatively simple, the actual behavior of the network depends on the gene transcription rates, with different transcription rates causing different static and dynamic outcomes [36]. The present study analyzes the crucial aspect of gene transcription control in a quantitative way, and characterizes a modular system for the control of transcription in gene networks. Moreover, the adopted gene circuit has a negative feedback structure, which is shared by a plethora of synthetic devices, and also plays a central role in diverse natural processes, from pattern formation to development. The negative feedback structure speeds up the time for the response of the gene network, and also stabilize its behavior [37]. Thus, the adopted architecture of the network could be a useful solution for gene transcription control in synthetic devices.

## Conclusions

Our results support a bottom-up approach to gene circuit design, but they also highlight the importance of characterizing the elementary blocks in the appropriate experimental conditions. If this is certainly a severe constraint for a bottom-up approach to gene circuit design, it does not rule out this possibility. Synthetic devices work by the interplay of several gene circuits, and usually the hard step in the design of new devices is to tune the transcription of different genes in order to get the desired output. If molecular interplays are shut down one-by-one - as it has been done here by removing the operator sequence in the *Reporter *plasmid - the elementary parts can be characterized in an isolated environment. Then, this information can be used to facilitate the design of new synthetic devices. Moreover, it is important to remember that cell transformation by a second plasmid is a strong test for the modular architecture. If changes are minimal perturbations for the biological system, the deviation from a perfect modularity will likely be smaller, and a functional characterization of the elementary parts in standardized experimental conditions could be adequate to help in the design of new gene circuits.

## Methods

### Plasmid construction

All the biological parts were taken from the Registry of Standard Biological parts, except the lactose operator sites that were synthesized (GeneArt) in the BioBrick standard format. The *Reporter *gene circuit was cloned in a high copy number plasmid (pSB1A2) containing Ampicillin resistance and a pUC19-derived pMB1 replication origin. The *Repressor *gene circuit was cloned in a medium copy number plasmid (pSB3K3) containing Kanamycin resistance and a pMR101-derived p15A replication origin. The constitutive promoters P_2547 _(BBa_J23100) and P_1429 _(BBa_J23118) were cloned upstream of the lactose operator sequences, respectively in the high and medium copy number plasmid. Three lactose operator sequences were used: O_1_, aattgtgagcggataacaatt; O_2_, aaatgtgagcgagtaacaacc; and O_s_, aattgtgagcgctcacaatt [27,38]. The GFP gene (BBa_J04031), with a LVA degradation tag, was placed downstream of the P_2547_O_x _regulated promoters in the high copy number plasmid. The LacI-coding sequence with LVA degradation tag (BBa_C0012) was placed downstream of the P_1429_O_x _regulated promoter in the medium copy number plasmid. The same ribosome binding site (RBS) sequence (BBa_B0034) was cloned upstream of all the protein coding sequences. A double transcriptional terminator T (BBa_B0015) was placed downstream of each transcriptional unit. Moreover, a double terminator is present at both sides of the plasmid multiple cloning site in order to prevent random transcriptions. In order to test the modular approach and to better identify the parameters of the mathematical model, we also built: (i) *Reporter *plasmids lacking the operator sequence; (ii) high and medium copy number plasmids both with a non-tagged GFP protein (BBa_I13504) cloned downstream of the P_1429 _promoter; (iii) *Reporter *plasmids with the P_1429 _promoter lacking the operator sequence.

### Fluorescence measurements

Dh5α *E. coli *cells were grown at 37°C in 5 ml of M9 minimal medium, supplemented with casamino acids, thiamine hydrochloride and the appropriate antibiotics. Glucose was the main carbon source (Sigma). Fluorescence measurements were performed after an overnight growth in presence of the desired amount of Isopropyl β-D-1-thiogalacto-piranoside (IPTG; Sigma). 100 μl samples were transferred from each cell culture into a multi-well plate and measured in a Wallac VICTOR^2 ^reader (Perkin Elmer). Both fluorescence (Fluo; Ex 501/Em 511 nm) and optical density (OD; 600 nm) were measured. Normalized fluorescence, [*GFP*]*au*, in arbitrary unit (au), was estimated as:

(2) $$[GFP]au=\frac{Fluo-Fluo_{DH5\alpha }}{OD},$$

where *Fluo *and *OD *are respectively the fluorescence and the optical density of the sample, and *Fluo*_*DH5*__α _is the average auto-fluorescence in samples of non-transformed Dh5α cells. These measurements, referred to as overnight growth, correspond to samples evaluated at time equal to 12 h, with an average OD of 0.36 ± 0.10. This time point was determined by preliminary time-course measurements (Additional File 1, Figure S1) where cells were grown up to 14 hours and sampling done every 30 min for different cell cultures; namely: (i) cells transformed with a single plasmid; (ii) cells co-transformed with two plasmids; (iii) cells co-transformed with two plasmids, and induced by IPTG. Throughout these measurements, normalized fluorescence reached a steady-state value after ~11 h, for all the tested cell populations, and this value was maintained stable for the following 3 hours measured. This steady state behavior justifies the static measurements used for the remaining analyses. The linear relationship between absolute fluorescence and optical density (Additional File 1, Figure S1) justifies the normalization procedure performed by equation 2.

### Statistical analysis

Values are reported as mean ± standard deviation. One-way ANOVA, and the Bonferroni post-hoc test for pairwise comparisons were used for detecting differences in normalized fluorescence between multiple groups, for which normal distributions were found (Jarque-Bera test). A significance level of 95% (p < 0.05) was used for all the statistical analyses. MATLAB package (2007a, The MathWorks, Natick, MA) was used for the statistical tests.

### Mathematical model and parameter definition

The O_x_O_y _gene-circuit - *Reporter *plasmid with the O_x _operator sequence and *Repressor *plasmid with the O_y _operator sequence - was described by the following differential equations:

(3) $$\frac{dM_{G/L}}{dt}=\alpha_{G/L}^{M}D_{G/L}^{F}-\lambda_{G/L}^{M}M_{G/L},$$

(4) $$D_{G/L}^{F}=D_{G/L}^{0}-D_{G/L}^{L}-D_{G/L}^{I},$$

(5) $$\frac{dD_{G/L}^{L}}{dt}=\frac{1}{\tau^{DL}}\left[\frac{D_{G/L}^{F}L^{F}}{K_{x/y}^{L}}-D_{G/L}^{L}\right],$$

(6) $$\frac{dD_{G/L}^{I}}{dt}=\frac{1}{\tau^{DI}}\left[\frac{D_{G/L}^{F}L^{I}}{K_{x/y}^{I}}-D_{G/L}^{I}\right],$$

(7) $$\begin{array}{l} \frac{dL^{I}}{dt}=\frac{1}{\tau^{LI}}\left[L^{F}{\left(\frac{I}{K^{LI}}\right)}^{n}-L^{I}\right]-\\ \;-\lambda_{L}L^{I}-\frac{dD_{G}^{I}}{dt}-\frac{dD_{L}^{I}}{dt}, \end{array}$$

(8) $$\begin{array}{l} \frac{dL^{F}}{dt}=\alpha_{L}M_{L}-\lambda_{L}L^{F}-\frac{1}{\tau^{LI}}\left[L^{F}{\left(\frac{I}{K^{LI}}\right)}^{n}-L^{I}\right]-\\ \;-\frac{dD_{G}^{L}}{dt}-\frac{dD_{L}^{L}}{dt}, \end{array}$$

(9) $$\frac{dG}{dt}=\alpha_{G}M_{G}-\lambda_{G}G.$$

Subscripts *G *and *L *are used for the variables/parameters of *Repressor *and *Reporter *gene-circuit respectively. Table 1 defines all the symbols used in equations 3-9. Differently from other mathematical models of the lactose repressor system, the equations adopted here take into account a possible inhibition of transcription by induced LacI molecules (*L*^*I*^, LacI molecules bound to IPTG). Each promoter switch among three possible states: promoter bound to LacI, $D_{G/L}^{L}$; promoter bound to induced LacI, $D_{G/L}^{I}$; and free-promoter, $D_{G/L}^{F}$ (Equation 4). As a comparison, simulations without residual affinity of induced lactose repressor molecules for the operator sites were also performed. The mathematical model used for these simulations is provided as supplementary material (Additional File 2). In brief, differential equations 6 and state variables $D_{G/L}^{I}$ were removed $\left(D_{G/L}^{I}\equiv 0\right)$. The parameters for the two mathematical models were the same, with except of the $K_{x/y}^{I}$ parameters that do not appear in the model without residual affinity.

The total number of *Reporter *plasmids per cell (high copy number plasmids, $D_{G}^{0}$), was assumed equal to 80. This value only scales the other parameters of the model, but not the conclusions drawn from equations 3-9. In order to estimate the ratio $D_{G}^{0}/D_{L}^{0}$, we measured the fluorescence in cells transformed with: (i) *Reporter *plasmid missing the operator sequence and with the non-tagged GFP protein (BBa_I13504) cloned downstream of the P_1429 _promoter, and (ii) the same gene circuit cloned in the medium copy number plasmid. Cloning the *Reporter *and *Repressor *circuits respectively on a high and medium copy number plasmid gives higher sensitivity to IPTG induction, allowing a more accurate quantitative analysis. At the same time - in the context of a modular approach to gene circuit design - the types of plasmids adopted can be treated as further interchangeable parts of the circuit. As described above, the ratio between the number of high and medium copy number plasmids per cell was estimated by a separate set of experimental measurements in simplified circuits, and then used for the analysis of all the other circuits - which is the typical approach used for the design of complex circuits using modular parts and a bottom-up strategy.

The degradation rate of mRNA, $\lambda_{G/L}^{M}$, was set to 1/3.69 min^-1 ^[39], both for LacI and GFP. Protein degradation rate, *λ*_*G/L*_, was measured experimentally by first inducing the O_1_O_1 _circuit with saturating concentration of IPTG, and then monitoring the GFP decay. In detail, after overnight grown in medium containing 1 mM IPTG, cells were washed with fresh medium and let at 37°C for growth in IPTG-free medium. Samples were taken every 15 minutes for fluorescence measurement. The GFP translation rate was set to 540*λ*_*G*_, in order to have a ratio of 540 between the number of mRNA and protein molecules at equilibrium [40]. Since the functional form of LacI is tetrameric, the LacI transcription rate was set to *α*_*L*_*= α*_*G*_/4. This choice preserves the ratio of 540 between the number of protein chains and mRNA molecules, assuming that this number is the characteristic gain of the translation process. The equilibrium binding constant of the complexes LacI-O_1 _($K_{1}^{L}$) and LacI-O_2 _($K_{2}^{L}$) were set to 0.133 and 1.63 molecule/cell respectively [41] (a cell volume of 1 μm^3 ^was assumed [42]). The value of the mRNA transcription rate constant for the *Reporter *circuit, $\alpha_{G}^{M}$, was estimated by fitting the relative induction of the O_1_O_1 _and O_2_O_2 _circuits in absence of IPTG (See the Results section for the definition of relative induction). The ratio $\alpha_{G}^{M}/\alpha_{L}^{M}$ was measured experimentally, comparing the cell fluorescence in cells transformed with *Reporter *plasmids with the P_2547 _or the P_1429 _promoter and without the operator sequence. Using a stronger promoter in the *Reporter *plasmids, compared to the *Repressor *plasmids, yields higher sensitivity to IPTG induction. As done for the ratio between the number of plasmids inside the cell, the strength-ratio between the two promoters was identified by experimental measurements in simplified circuits, and then used for the analysis of the remaining gene-circuits, with a typical modular approach to gene circuit design.

The value of the equilibrium binding constant for the LacI-O_S _complex ($K_{s}^{L}$) was defined by fitting the relative induction of the O_S_O_S _circuit in absence of IPTG. The constants for the binding of induced LacI molecules to the three operators sites ($K_{1}^{I}$, $K_{2}^{I}$ and $K_{s}^{I}$) and the parameter for the LacI-IPTG binding (*n *and *K*^*LI*^), were defined by fitting the experimental dose response curves of the three circuits O_2_O_2_, O_1_O_1 _and O_s_O_s_. A separate fitting procedure of the parameters *n *and *K*^*LI *^for the mathematical model without residual affinity between induced lactose repressors and operator sites was also performed, but it did not improve the agreement with the experimental data. Indeed, the model without residual affinity cannot reproduce dose-response curves with a maximum *GFP*_% _below 100%. Fitting procedure were performed by the *fminsearch *routine of the MATLAB package (2007a, The MathWorks, Natick, MA) minimizing the percentage error.

## Competing interests

The authors declare that they have no competing interests.

## Authors' contributions

FC built all the genetic constructs used in this work and performed all the experimental measurements. SF developed the mathematical model and performed the fitting procedure and parameter identification. EG advised on wet-lab procedures. SC designed the experiments conducted by FC and the model developed by SF. All authors wrote, read and approved the final manuscript.

## Supplementary Material

Additional File 1 **Preliminary dynamical measurements**. Time-course measurements of normalized fluorescence and optical density in cells transformed with different gene-circuits. Click here for file

Additional File 2 **Mathematical model without induced LacI residual affinity**. Equations of the mathematical model without residual affinity between induced lactose repressor molecules and operator sites Click here for file
