# Supplementary material for: Rational design of modular circuits for gene transcription: A test of the bottom-up approach
Source: J Biol Eng. 2010 Nov 11;4:14. doi: 10.1186/1754-1611-4-14 (PMC2993646; doi:10.1186/1754-1611-4-14)
Supplement: Additional File 1 — Preliminary dynamical measurements. Time-course measurements of normalized fluorescence and optical density in cells transformed with different gene-circuits. [file 1754-1611-4-14-S1.PDF]

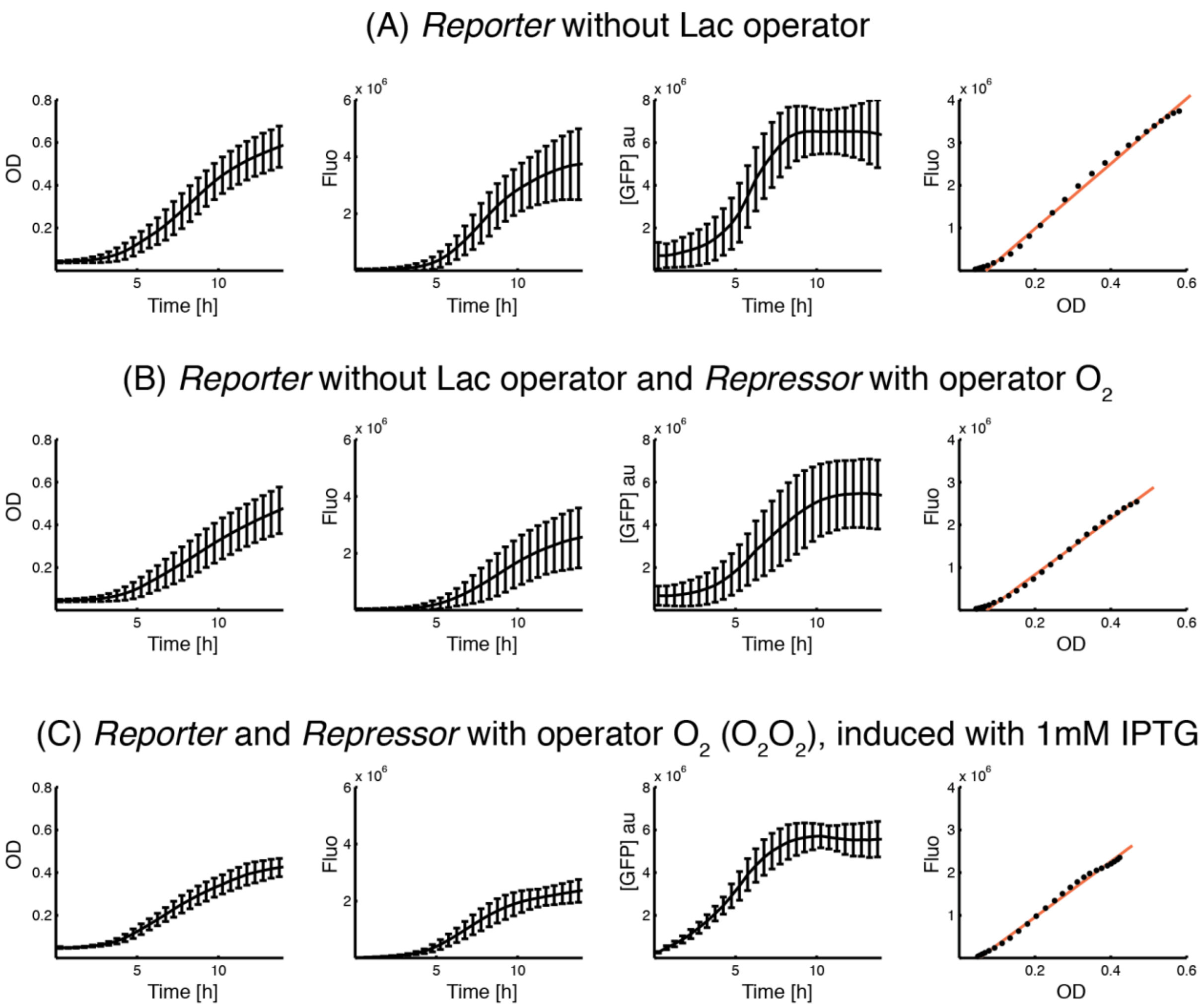

Figure S1. Dynamical behavior of cells transformed with: (A) *Reporter* plasmid without the operator site; (B) *Reporter* plasmid without the operator site, and *Repressor* plasmid with the operator site O_2; (C) *Reporter* and *Repressor* plasmids both with the operator site O_2, induced with 1 mM IPTG. The mean values and standard deviations in at least 10 separate measurements are shown, for the optical density value (first column), the absolute fluorescence (second column), and the normalized fluorescence (third column). The absolute fluorescence values are reported in the arbitrary units provided by the multi-well Wallac VICTOR[2]

reader (Perkin Elmer). In the fourth column the absolute fluorescence (average values) is shown as a function of the optical density (average values). The interpolating lines were defined by minimizing the root-mean-square distance from the experimental points with an OD value above 0.1.
