# Supplementary material for: Rational design of modular circuits for gene transcription: A test of the bottom-up approach
Source: J Biol Eng. 2010 Nov 11;4:14. doi: 10.1186/1754-1611-4-14 (PMC2993646; doi:10.1186/1754-1611-4-14)
Supplement: Additional File 2 — Mathematical model without induced LacI residual affinity. Equations of the mathematical model without residual affinity between induced lactose repressor molecules and operator sites [file 1754-1611-4-14-S2.PDF]

Mathematical model without residual affinity between induced lactose repressor molecules and operator sites

$$\frac{dM_{G/L}}{dt} = \alpha^{M}_{G/L} D^{F}_{G/L} - \lambda^{M}_{G/L} M_{G/L} \qquad \text{(S1)},$$

$$D^{F}_{G/L} = D^{0}_{G/L} - D^{L}_{G/L} \qquad \text{(S2)},$$

$$\frac{dD^{L}_{G/L}}{dt} = \frac{1}{\tau^{DL}} \left[\frac{D^{F}_{G/L} L^{F}}{K^{L}_{x/y}} - D^{L}_{G/L} \right] \qquad \text{(S3)},$$

$$\frac{dL^{I}}{dt} = \frac{1}{\tau^{LI}} \left[L^{F} \left(\frac{I}{K^{LI}} \right)^{n} - L^{I} \right] - \lambda_{L} L^{I} \qquad \text{(S4)},$$

$$\frac{dL^{F}}{dt} = \alpha_{L} M_{L} - \lambda_{L} L^{F} - \frac{1}{\tau^{LI}} \left[L^{F} \left(\frac{I}{K^{LI}} \right)^{n} - L^{I} \right] - \frac{dD^{L}_{G}}{dt} - \frac{dD^{L}_{L}}{dt} \qquad \text{(S5)},$$

$$\frac{dG}{dt} = \alpha_{G} M_{G} - \lambda_{G} G \qquad \text{(S6)}.$$
